# Novel Grafted/Crosslinked Cellulose Acetate Membrane with *N*-isopropylacrylamide/*N,N*-methylenebisacrylamide for Water Desalination

**DOI:** 10.1038/s41598-020-67008-3

**Published:** 2020-06-18

**Authors:** Yasmeen Elkony, El-Sayed Mansour, Amel Elhusseiny, Hammed Hassan, Shaker Ebrahim

**Affiliations:** 1 Chemistry Department, Faculty of Science, Alexandria University, P.O.Box 426-Ibrahimia, 21321 Alexandria, Egypt; 2 Department of Materials Science, Institute of graduate studies and research, Alexandria University, Alexandria, Egypt

**Keywords:** Engineering, Chemical engineering, Polymers, Characterization and analytical techniques, Process chemistry, Biopolymers, Supramolecular polymers

## Abstract

This work aims to prepare new types of grafted and crosslinked cellulose acetate (CA) reverse osmosis (RO) membranes by phase inversion technique. The grafting and/or crosslinking processes of the pristine CA-RO membrane were conducted using *N*-isopropylacrylamide (N-IPAAm) and *N,N*-methylene bisacrylamide (MBAAm), respectively. The grafting/crosslinking mechanism onto the CA-RO membrane surface was proposed. Atomic force microscope (AFM) images of the pure CA-RO and 0.1 wt% N-IPAAm-grafted CA-RO membranes revealed that the surface roughness was 42.99 nm and 11.6 nm, respectively. Scanning electron microscopy (SEM) images of the 0.1 wt% grafted/crosslinked membrane indicated the finger-like macrovoids structure. It was observed that the contact angle of the pristine CA-RO membrane was 66.28° and declined to 49.7° for 0.1 wt % N-IPAAm-grafted CA-RO membrane. The salt rejection of the pristine CA-RO membrane was 93.7% and increased to 98.9% for the grafted 0.1 wt % N-IPAAm/CA-RO membrane. The optimum grafted/crosslinked composition was 0.1 wt %/ 0.013 wt % which produced the salt rejection and water flux of 94% and 3.2 L/m^2^h at low pressure, respectively. It was concluded that both the grafting and crosslinking processes enhanced the performance of the CA-RO membranes.

## Introduction

The RO membranes process is one of the most reliable, efficient and cost-effective water desalination techniques to meet the demand and indigence of the fresh water^1–3^. The most common polymeric materials used in RO membrane manufacture are either cellulose acetate (CA) or polysulfone coated with dense layer of polyamide thin film^4–7^. Surface functionalization enables the RO membranes to have a better water quality, stability and permeability and consequently reducing RO operational costs and energy consumption. The surface properties of RO membranes, including hydrophilicity, water permeability, salt rejection, water flux, and surface roughness are the main parameters which determine water affinity and the interactions mechanism with salts^8^.

Surface modification techniques such as grafting by hydrophilic molecules have been made to improve the membrane performance^9,10^. Different types of the graft polymerization of monomers onto cellulose were carried out by radical, ionic and ring opening or living radical polymerizations^11–15^. The modification of the membrane surfaces can be carried out by “grafting-from” and “grafting to” approaches. In the grafting-to method end-functionalized polymer molecules bind with complementary functional groups located on the surface to produce tethered chains. However, this method is rarely to be used in the membrane modification because of the low density of grafted polymer chains. The grafting from technique uses the polymerization initiated from the substrate surface by attached initiating groups. Molecules of a monomer penetrate through the already grafted polymer layer easily and significant grafted amounts can be reached. This technique is commonly used for the preparation of thick grafted layer with high grafting density^16^.

Polymeric materials based on N-isopropyl acrylamide (N-IPAAm) and N,N-diethyl acrylamide (DEAAm) monomers are used as thermosensitive hydrogels. Fabrication of N-IPAAm-modified membrane showed good water permeability at low temperatures and have been used in gel actuators^17–19^. The thermoresponsive groups and copolymers were grafted onto the surfaces of cellulose to prepare the thermoresponsive membrane for efficient separation^19–21^. The addition of surfactants in aqueous/organic solution was studied in the *in situ* polymerization of thin film composite membranes^22^.

With respect to graft from there are also two main types. Firstly, atom transfer radical polymerization (ATRP) which is suited to grafting from CA surfaces, since free hydroxyl groups at the substrate surface can be converted to ATRP initiators, and subsequently used to initiate the growth of polymer brushes. However, the disadvantage of using ATRP in a manufacturing process is that it requires inert conditions and a large amount of copper catalyst, which is difficult to remove from the final product^23,24^. In addition, this method requires multiple steps, and this leads to an inevitable loss of a small amount of reactive polymer chains and formation of polymeric impurities^25^. Secondary, the redox initiated grafting offers a possibility to modify membrane surfaces *in situ*. A redox system composed of potassium persulfate was used to generate initiating radicals for graft polymerization of acrylic acid, N-isopropylacrylamide, and other hydrophilic monomers at the surface of CA membranes. The disadvantages of this method and these monomers are the blocking of the pores of support CA membrane due to the taking place of the polymerization inside these pores, particularly for high degrees of grafting and consequently the flux will be decreased^26^.

Temperature sensitive poly (N-isopropylacrylamide) (PNIPAAM) was grafted via *in situ* and *ex situ* free radical polymerization onto CA ultrafiltration membrane. However, the flux measurements did not support the ability of the NIPAAM-modified membranes to respond to temperature activations since the membranes did not show any improvements when operated under a temperature cycle^27^. Free-radical graft polymerization of 3-allyl-5,5- dimethylhydantoin and then crosslinked by N,N′-methylenebis(acrylamide) were performed onto the commercial polyamide RO membrane to enhance the chlorine resistance and water flux^28^.

To the best of our knowledge the preparation of N-IPAAm-grafted CA-RO membrane has not previously reported. In this study modified cellulose acetate-based membranes (CA-g-N-IPAAm) are synthesized by grafting N-IPAAm with different ratios onto cellulose acetate membranes via free radical polymerization. The relatively most efficient grafted membrane was further treated with different concentrations of the crosslinker MBAAm to enhance the membrane performance. The prepared membranes were tested in a cross flow RO cell by applying different operating pressures to measure the salt rejection and water flux. The chemical structures of the prepared membranes were characterized by Fourier transforms infrared (FTIR) spectroscopy and the postulated grafting/crosslinking reaction mechanism of the resulting cellulose-based material is proposed. Moreover, the surface morphology is investigated and studied by SEM and AFM.

## Materials and Methods

### Materials

1,4-Dioxane was supplied by Panreac Quimica S.A (Barcelona, Spain). The acetic acid (purity > 99.8%) was obtained from BDH Analar (England). Methanol (purity > 99.5%) and acetone (purity > 99%) were received from Labsolve (Lisbon, Portugal). Ethanol was delivered from Carlo Erba. Cellulose acetate (molecular weight of 100,000 g/mol and 39.8 wt % acetyl) was obtained from Aldrich. N-isopropylacrylamide and N,N-methylene bisacrylamide were purchased from Across (New Jersy, USA). Potassium persulfate (KPS), NaOH were received from Sigma Aldrich. NaCl was supplied by Honeywell, (Denmark).

### Preparation of pristine CA-RO membrane

The CA-RO membrane was prepared by phase inversion technique and following the procedure reported in the literature^5^. The casting solution was prepared by mixing CA powder with several solvents containing (CH3)2CO/AcOH/dioxane/CH3OH) (11: 5.5: 1: 2). This solution was cast on a glass plate surface and a proper thickness was adjusted at 250 µm by using a casting knife of the automatic film applicator (Zehntner 2300-Swiss) at a constant speed of 10 mm/s. The membrane was left to evaporate the solvent for 60 seconds and then immersed in a coagulation water bath at 4 °C for 15 min. The membrane was then transferred to another cold-water bath at the same temperature for 2 h. Finally, the membrane was annealed in water bath at 60 °C and was gradually increased to 80 °C for 10 min.

### Grafting of CA-RO membranes

The plate CA-RO membrane and gasket frame stack were held together by binder clips. The partially deacetylation of the surface of CA-RO membrane was set by pouring 0.014 wt % of NaOH onto the surface of the membrane for 2 min to facilitate the grafting process then the surface was washed several times with deionized water. Potassium persulfate (1.5%) was added dropwise on the surface and left for 10 min to ensure the reaction completion and formation of the free radicals on whole the membrane surface area^29^. The growth of graft chains was carried on these hydrogen-abstracted active sites. The resulted activated membranes were separately poured in different concentrations of N-IPAAm/water for 10 min at room temperature. Residual droplets of the grafting solution were squeezed off using soft rubber roller to ensure that no visible aqueous droplets may form defects. Finally, the grafted membranes were treated in an oven at 40 °C for 10 min^27–30^.

### Crosslinking of grafted CA-RO membranes with MBAAm

The relatively efficient 0.1% N-IPAAm/CA membrane was selected for further treatment as described above with different concentrations of the crosslinker MBAAm in 5% aqueous ethanol for 10 min and then thermally treated in an oven at 40 °C for 10 min to obtain four grafted crosslinked membranes.

### Characterization techniques and RO membrane performance measurement

The chemical structure of the prepared membranes was characterized by FTIR spectroscopy (FTIR-8400S Shimadzu in the range between 4000 and 400 cm^−1^. The morphology of the pure, the grafted and grafted/crosslinked membranes were studied using SEM, XL 5300 JEOL. The cross sections of the RO membranes were prepared through cutting off under liquid nitrogen for a consistent and clear break cross section imaging and have been coated by golden thin film using sputter coating technique. AFM images were captured by taken the membrane samples with dimensions of 1×1 µm, using SPM-9700 Shimadzu equipped with dynamic mode at ambient temperature. A microcantilever OMCL-TR800PSA (Olympus) was used. The contact angles have been measured to investigate the surface hydrophilicity of the membranes using a Rame hart, Instrument Company, France. A droplet of distilled water (2 μL) was dropped on the RO membrane surface (3 cm ×2 cm) using microsyringe Hamilton Company, Reno, NV.

The salt rejection and water flux were conducted for RO membranes using a cross flow RO unit that was purchased from Sterling Company (CF042). The RO cell consists of hydraulic pump, pressure gauge, pressure control valve, flow meter and variable frequency drive. Saline salt solution of NaCl of 10,000 ppm at pH 7 was used to simulate brackish water. Before the permeation tests, the compaction of the membranes was carried out a constant operating pressure with deionized water for 1 h to obtain a steady flux and ensure the stability of the RO membrane. The total dissolved salt of the permeate water was measured by a pH/conductivity meter 430 portable, Jenway, England.

The flux J_V_ was calculated using Eq. (1)^31^.

1 $${\rm{Jv}}={\rm{V}}/{\rm{At}}$$

where V is the permeate volume per unit area (A/m^2^) per unit time (t/h).

The value of rejection (R) was calculated using the Eq. (2)^32^.

2 $$R=(1-\frac{Cf}{{\rm{Cv}}})\times 100$$

where *C*_*v*_ is concentration of solute in permeate, *C*_*f*_ is the concentration in the feed.

## Results and Discussion

### Mechanism of grafted/crosslinked CA-RO membrane

**Figure 1:**
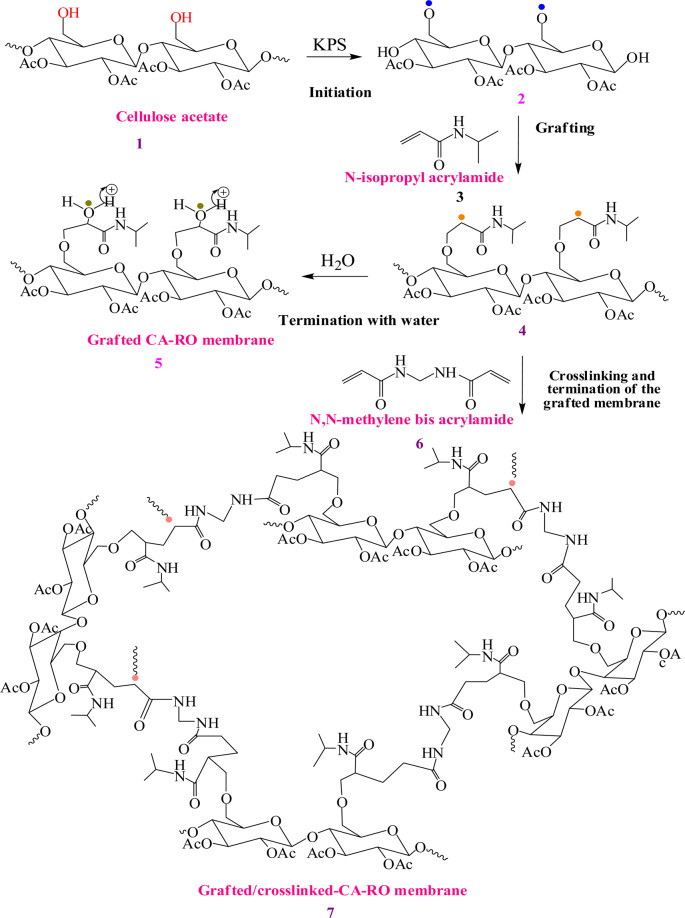
Proposed grafting of CA membrane with N-IPAAm and/or the radical crosslinking with N, N-methylene bisacrylamide.

As shown in Fig. 1, grafting of CA-RO membrane **1** is conducted by reaction with different concentration of the hydrophilic monomer N-IPAAm **3** at in the presence of KPS to obtain the grafted CA/RO membranes **2**. Firstly, the surface of the CA-RO membrane was treated with 0.014 wt % NaOH for partially deacetylation to facilitate the polymerization on the surface of the membrane **1**. Secondly, the surface was initiated using KPS initiator to generate free radicals on the surface of the CA-RO membrane **2** could be attributed to the abstraction of hydrogen atom of the free C_6_-OH group due to its reactivity as a primary alcohol^5,33^. The propagation step started when the *in situ* radical terminals are added to vinyl terminal of N-IPAAm monomer to furnish the targeted intermediate **4**. Portion of the latter intermediate **4** is quenched with water to terminate the reaction and produces the grafted CA/RO membrane **5**, while the rest of **4** is further treated with different concentrations of MBAAm **6** to produce the targeted grafted/crosslinked CA-RO membrane **7** by creating a new link between the two monomers. The addition of the hydrophilic monomer N-IPAAm introduces polar functional groups to the surface of the membrane and improves the hydrophilicity. The grafting process could be influence the performance of the CA-RO membrane by enhancing the salt rejection and water flux because it forms a new hydrophilic layer with good coverage to the surface to enhance the dense layer properties and treat any defects on the membrane surface. To avoid the probability of the monomer penetration or blocking of the pores which decreases the flux of the membrane by grafting, the crosslinker was inserted to increase the interchain between polymer chain and formed a net structure^28^.

### Structural property of the grafted CA-RO membrane

**Figure 2:**
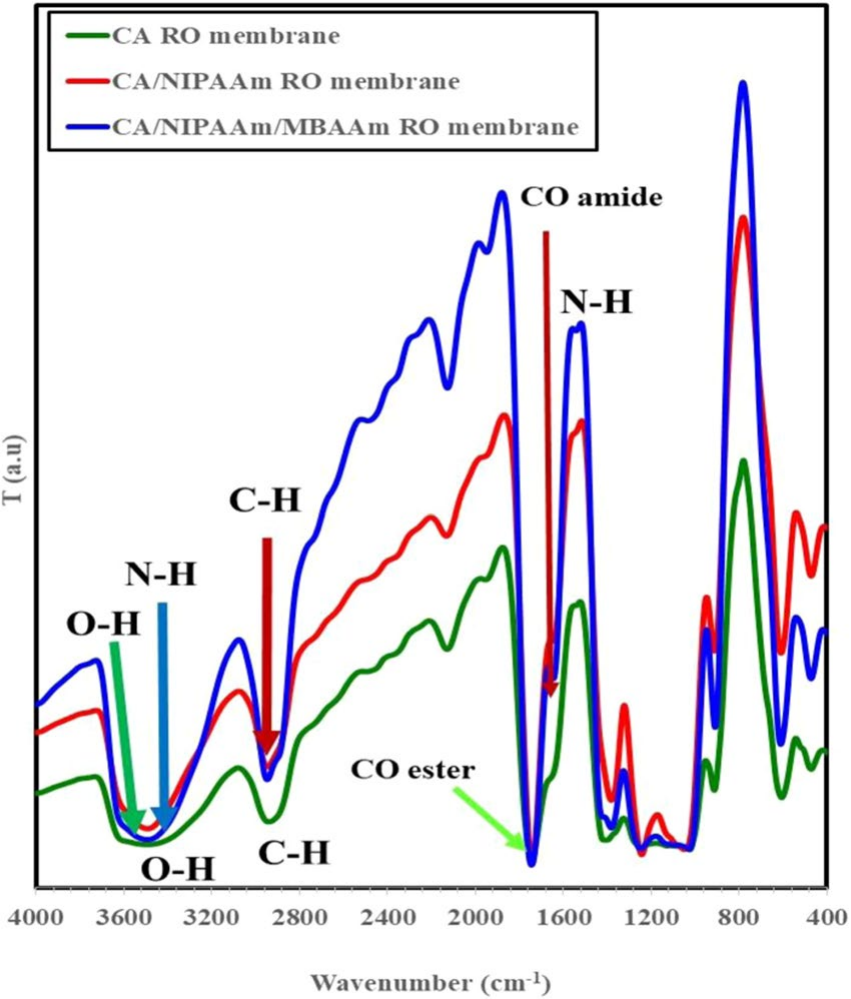
FTIR spectra of pristine CA-RO, 0.1 wt % N-IPAAm/CA-RO and 0.1 wt % N-IPAAm/ 0.013 wt % MBAAm membranes.

The FTIR spectra of CA-RO, grafted CA-RO and grafted/crosslinked CA-RO membranes are presented in Fig. 2. The FT-IR spectrum of the CA-RO membrane exhibits absorption bands at 3475 cm^−1^, corresponds to OH_str_, at 2941 cm^−1^ attributes to the H-C groups, at 1739 cm^−1^ belongs to CO ester group, and at 1257 cm^−1^ due to the C-O-C symmetric ether bond as illustrated in Fig. 2. The grafted CA-RO membrane absorption band at υ 1649 cm^−1^ is attributed to the CONH amide, at 1550 cm^−1^ corresponds to N-H bending, a broad band at 3500 cm^−1^ is belonged to OH and N-H _str_ and a band at 2953 cm^−1^ is due to the H-C groups^12,34^. On the other hand, the grafted/crosslinked membrane displays two successive sharp absorption bands at 1743 cm^−1^ and 1747 cm^−1^, correspond to the CO group as shown in Fig. 2. Noteworthy, the intensity of these bands is increased with increasing grafting agent concentration. The absorption bands at 1649 cm^−1^, 1537 cm^−1^ and 1404 cm^−1^ correspond to the CO amide, NH _bending_ and CN amide bands, respectively^11,12^.

### Morphological studies

**Figure 3:**
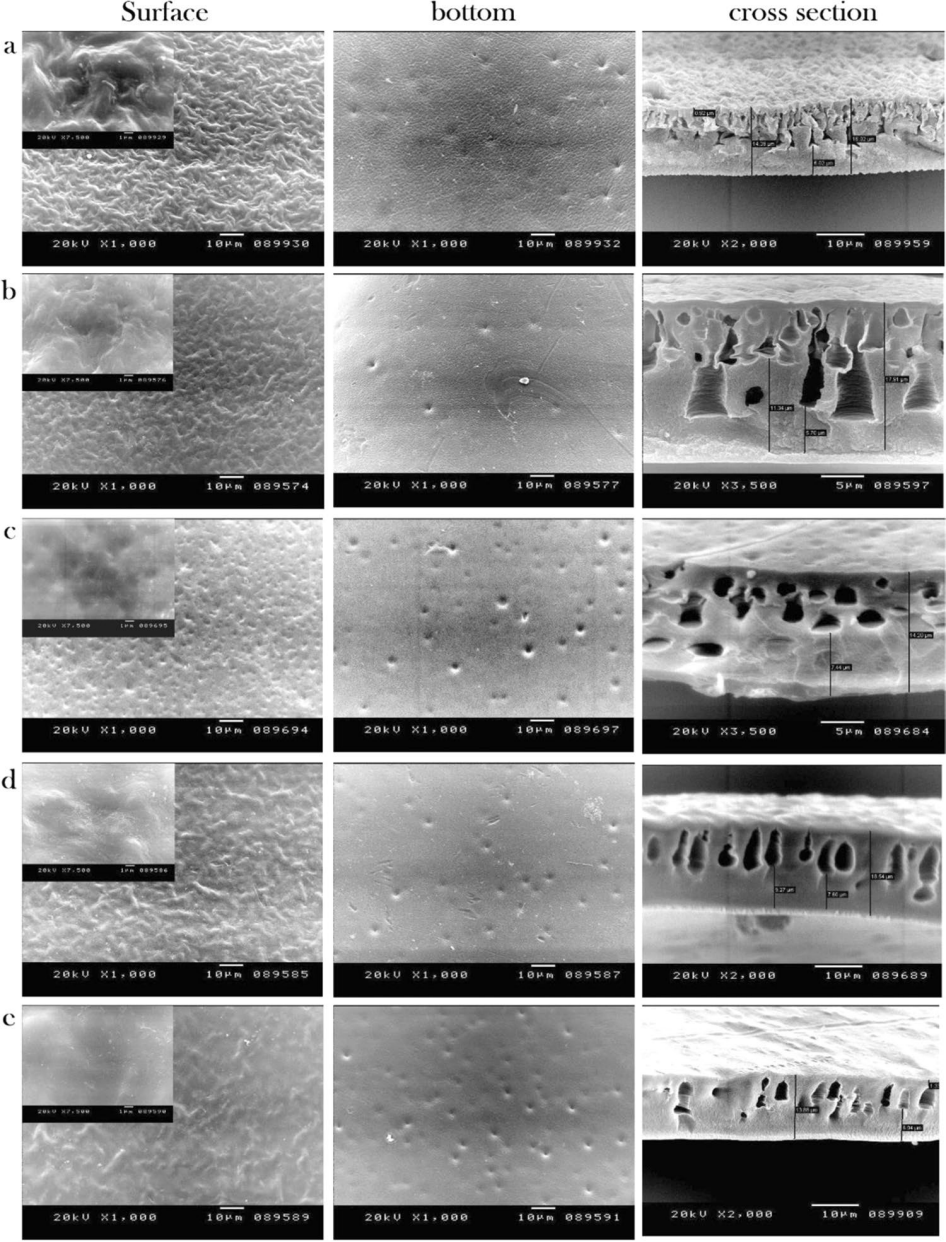
SEM images of surface, bottom and cross section: (**a**) CA-RO membrane, (**b**) 0.05 wt %N-IPAAm/CA, (**c**) 0.1 wt % N-IPAAm/CA, (**d**) 0.2 wt % N-IPAAm/CA, (**e**) 0.3 wt %N-IPAAm/CA-RO membranes.

The morphologies of the surface, bottom and the cross section of the CA-RO membrane and grafted 0.05%-, 0.1%-, 0.2%- and 0.3 wt %-N-IPAAm membranes are dispalyed in Fig. 3. The SEM image of CA-RO membrane shows a dense top layer with ridge, valley shape and porous sublayer as presented in Fig. 3(a). However, the cross-section photograph has small voids and large portion sponge-like structure, indicating the highly viscous casting solution^35^. The SEM images of the grafted CA-RO membranes using the above-mentioned grafting concentrations are shown in Fig. 3(b–e). The images depict denser skin top layers which increase from 0.92 µm of the pristine CA membrane to 1, 1.2, 1.3 and 1.33 µm, respectively for the different N-IPAAm concentrations of 0.05, 0.1, 0.2 and 0.3 wt % with smoother top surfaces than the pristine. Noteworthy, the ridge and valley shape are decreased and a high porosity with better pore shape and density is observed with increasing the grafting concentration.

The cross-section images of the grafted samples exhibit interesting morphologies. The cross sections have also asymmetric structures with dense top layers and channel or macrovoids like structures. For instance, the SEM image of a 0.05 wt % sample shown in Fig. 3(b) displays a channel-like structure, while the 0.1 wt % membrane (Fig. 3c) displays two layers of macrovoids and sponge like structure. Further increasing of the grafting agent concentrations to 0.2 wt %, and 0.3 wt% affords macrovoids layer morphologies as presented in Fig. (3d,e).

The bottom surfaces illustrate porous structures and the number of pores is enhanced and increased with the excess of the grafting concentration. These pores work as a water pump, while the fingers and macrovoids work as cannels. Consequently, if these pumps are blocked the water will not pass through the cannels and the pores of the bottom layer are the dominant parameter which will influence on the water flux.

**Figure 4:**
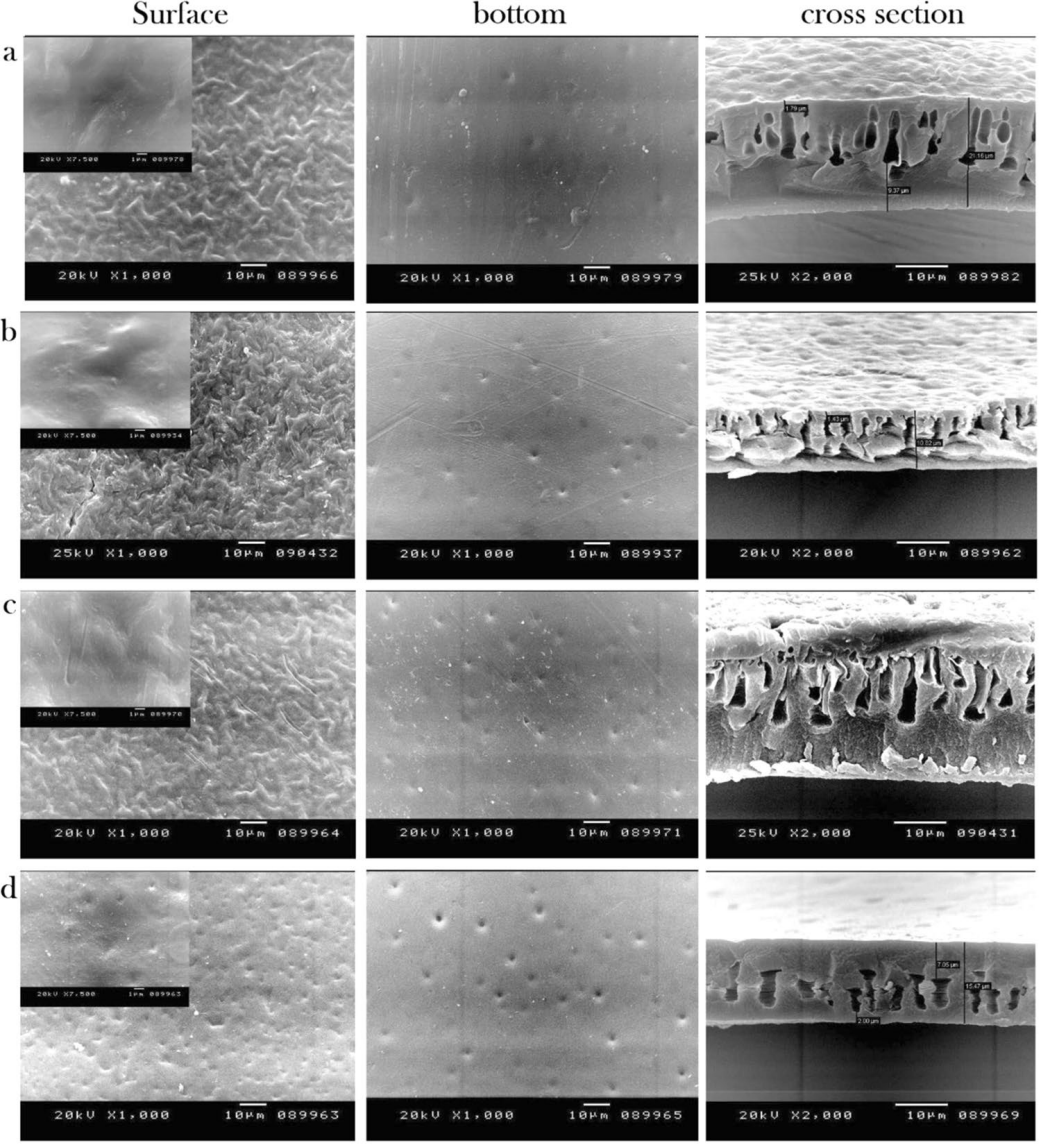
SEM images of surface, bottom and cross section: (**a**) CA /N-IPAAm/MBAAm (0.1:0.006 wt %), (**b**) CA / N-IPAAm / MBAAm (0.1:0.01 wt %), (**c**) 0.1 wt % CA /N-IPAA / MBAAm (0.1:0.013 wt %), (**d**) CA / N-IPAAm / MBAAm (0.1:0.02 wt %)/RO membranes.

The 0.1 wt % N-IPAAm grafted CA-RO membrane was selected for further crosslinking and morphological studies. The crosslinking process is conducted using 0.006 wt%, 0.01 wt%, 0.013 wt% and 0.02 wt% of the crosslinker MBAAm. The SEM images exhibits higher thickness of denser top layer than the grafted membranes in the range from 1.7 to 2 µm from 0.006 to 0.02 wt % of MBAAm. The cross-section images shown in Fig. 4(a–d) clearly indicate the formation of finger-like macrovoids structure. Noticeably, larger numbers of the macrovoids are observed for the 0.013 wt % MBAAm sample (Fig. 4c). Further rising of the crosslinking concentration up to 0.02 wt % furnishes the tight and lower voids structure as demonstrated in Fig. 4(d).

**Figure 5:**
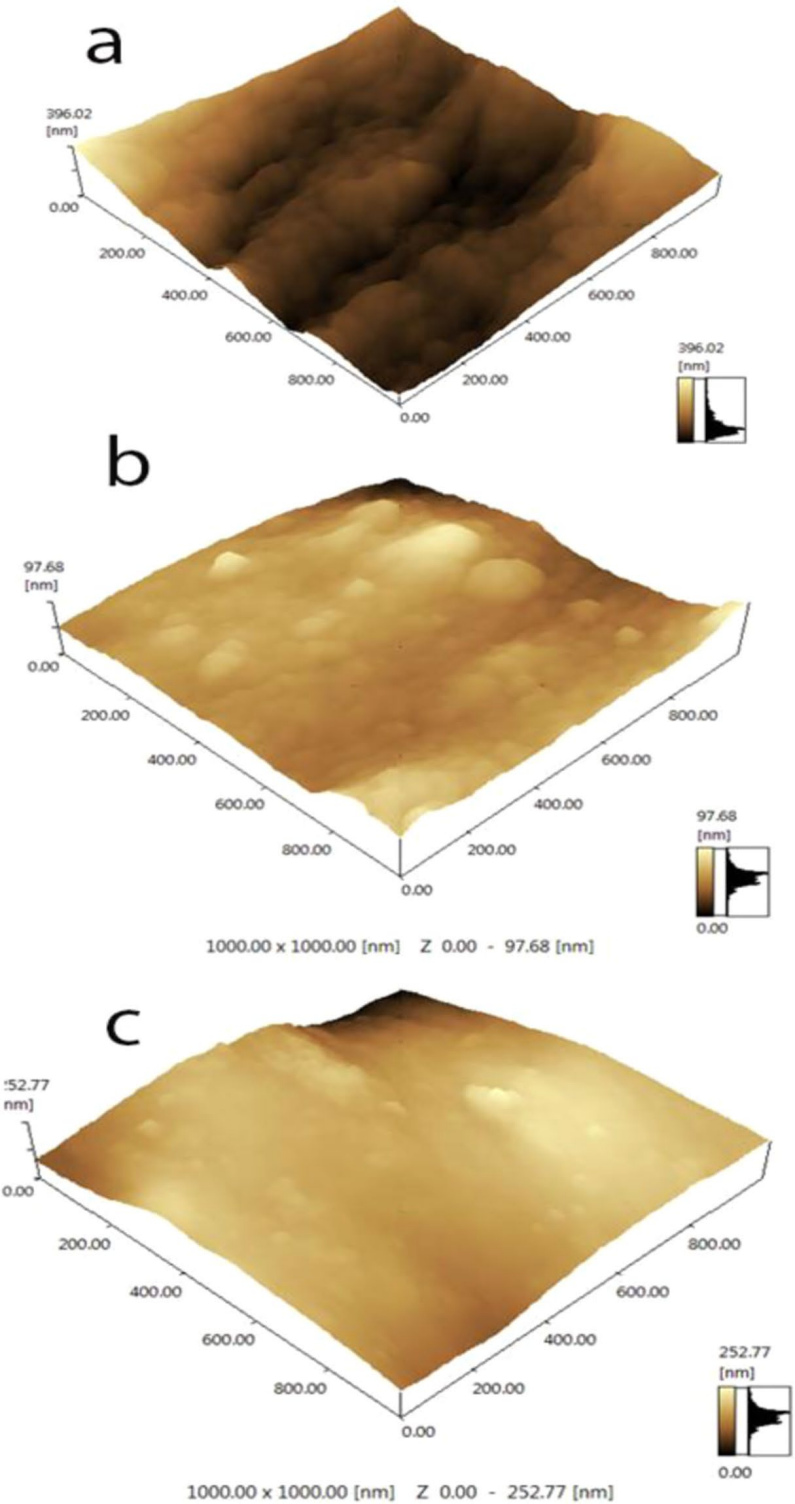
AFM images of: (**a**) CA-RO (**b**) 0.1 wt % N-IPAAm grafted- and (**c**) 0.1 wt % N-IPAAm grafted- /0.013 wt % MBAAm crosslinked membranes.

For structure-morphology correlation purpose, selected samples of CA-RO, 0.1 wt % N-IPAAm-grafted CA-RO and 0.1 wt % N-IPAAm grafted-/0.013 wt% MBAAm crosslinked CA-RO membranes are subjected to AFM scan area studies. The bright and dark regions are the peaks and valleys, respectively. R_a_ represents the average roughness and Rz is the difference in height between the average of the five highest peaks and the five lowest valleys along the assessment length of the profile^36^. R_a_ of the CA-RO membrane is 42.99 nm as shown in Fig. 5(a) which decreases to 11.6 nm for the grafted one (Fig. 5b) and to 27.1 nm in case of the grafted/crosslinked membrane. These results can be explained based on the grafted and grafted/crosslinked hydrophilic monomers fill the pores onto the surface and covered any defects and become more homogenous. Consequently, it is expected that the fluxes of the grafted and grafted/crosslinked RO membrane are declined. Whereas the salt rejection will be improved. Generally, the CA-RO surface roughness is susceptible to fouling compared to smooth surface^37^. Compared to the surface roughness of grafted CA-RO samples, the grafted/crosslinked CA/RO samples exhibit higher roughness than the grafted. This is attributed to the lower solubility of the crosslinker on the CA surface. Moreover, these results are attributed to the formation of additional network structure linked between the polymers chains which could be affected the smooth of the surface and formation of peaks and valleys more than the grafted monomer. Table (1) shows R_a_ and R_z_ values of the pristine CA, grafted CA and grafted/crosslinked CA-RO membranes. The grafted membrane presents a lower R_a_ and R_Z_ compared to the pure CA-RO membrane. On the other hand, the crosslinking increases the R_a_ and R_z_ values compared to the grafted CA-RO membranes.

**Table 1:** R_a_ and R_z_ of CA-RO, 0.1 wt % N-IPAAm grafted- and 0.1wt % N-IPAAm grafted/0.013 wt % MBAAm crosslinked membranes calculated by AFM (area 1×1 μm^2^).

AFM parameters	R_a_ (nm)	R_z_ (nm)
CA-RO Membrane		
Pristine CA-RO	42.99	355
Grafted CA-RO	11.16	101
Grafted/crosslinked CA-RO	27.1	229

### Effect of grafting on the hydrophilicity of grafted CA-RO membranes

**Figure 6:**
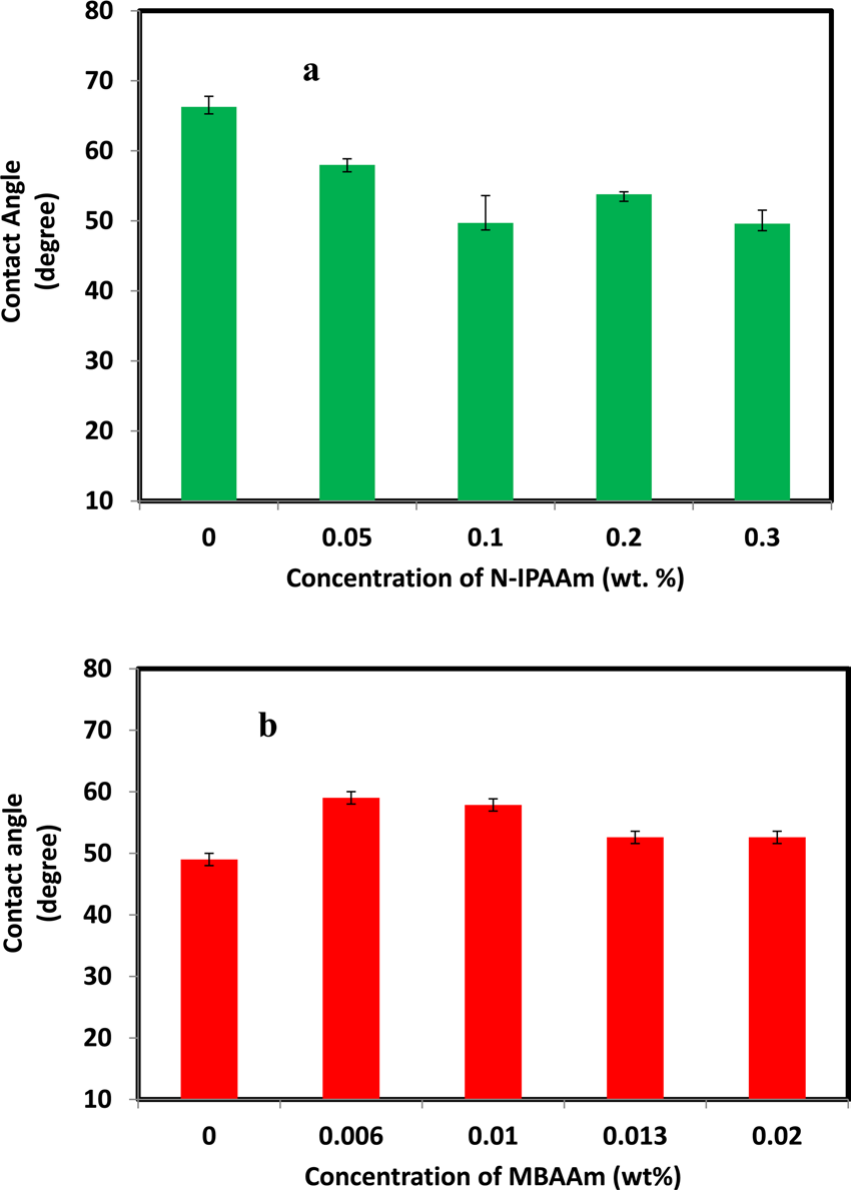
Contact angles of (**a**) grafted and (**b**) grafted/crosslinked CA- RO membranes.

The contact angle is a measurement of the hydrophilicity of the membrane surface. Higher hydrophilicity reduces the fouling and vice versa. The contact angle is measured by placing a microliter droplet of distilled water on the membrane surface. The angle is determined for both sides of the droplet and the mean value is then calculated. The contact angles versus concentration change of the grafting and/or crosslinking agents are presented in Fig. 6. From Fig. 6(a), the contact angle of pristine CA-RO membrane is 66.28°. This value is decreased to 58°, 49.7°, 53.8° and 49.6° by increasing the grafting concentrations to 0.05 wt%, 0.1 wt%, 0.2 wt % and 0.3 wt %, respectively. Interestingly, the contact angles of samples measured upon addition of different crosslinker concentrations of 0.006 wt%, 0.01 wt%, 0.013 wt%, and 0.02 wt% to the 0.1 wt % N-IPAAm grafted membrane are 59.02°, 57.85°, 52.6° and 52.6°, respectively. It is observed that the contact angle of the crosslinked CA-RO membrane is increased with respect to the grafted CA-RO membrane as a reference from 49.7° to 59. 02° for 0.006 wt % of the crosslinker. This is attributed to the raising of the roughness of the crosslinked RO membrane. Furthermore, increasing the crosslinker concentrating to 0.013 and 0.02 wt % increases the polar functionality of the surface and, thus improves the hydrophilicity to 52°. The hydrophilicity and hydrophobicity of the RO membrane are controlled by the electrostatic and/or hydrogen-bond interactions between the water molecules and functional groups. Because of the strong hydrogen-bond interactions between the water molecules and the surface functional groups, the RO-membrane affinity to water molecules is strong and the water droplet is spread on the membrane surface. The improvement of the hydrophilic properties for the grafted and crosslinked RO membrane is due to the presence of hydrophilic atoms (O and N) increases the wettability of the membrane. These groups form hydrogen bonds with the water molecules.

### Salt Rejection and water flux of untreated CA-RO membranes

**Figure 7:**
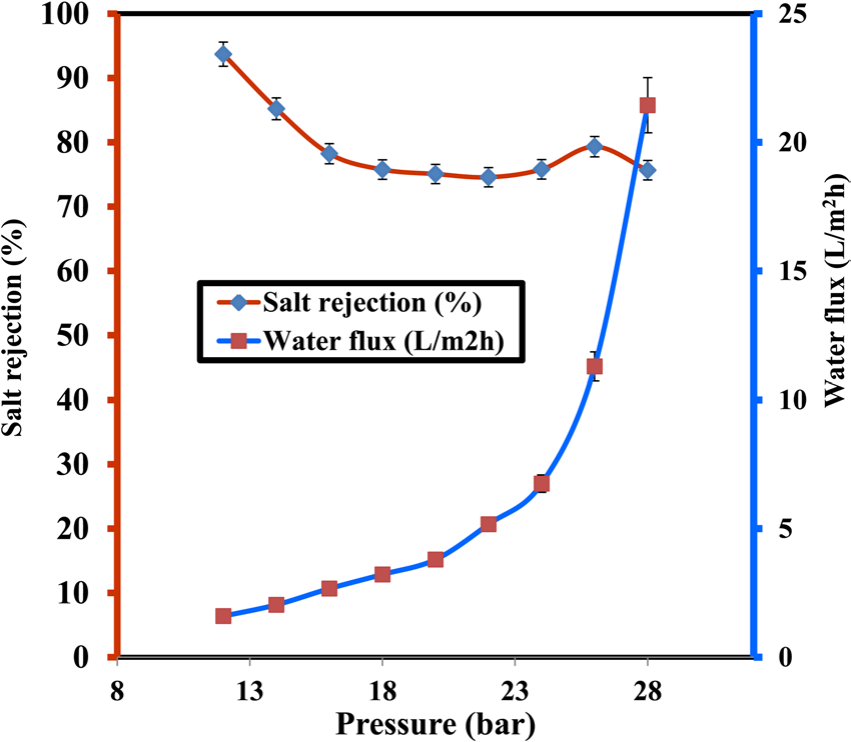
Salt rejection and water flux versus feed pressure of CA-RO membranes.

A testing RO cell was used to measure the flux and salt rejection values of the untreated CA-RO membrane at different operating pressures as represented in Fig. 7. It is found that the salt rejection of CA-RO membrane is 93.7% at 12 bar and decreases to 74.5 at 22 bar. The rejection decline is referred to the concentration polarization which formed boundary NaCl layer close to the surface and thus, the observed rejection of the feed bulk concentration is lower than the real rejection of the solute concentration. The permeate water flux of this membrane is 1.6 L/m^2^h at 12 bar and increases to 5.17 L/m^2^h at 22 bar. Hence, it is concluded that the water flux is increased proportionally with increasing the operating pressure and is matched with the solution diffusion model^38^.

### Salt rejection and water flux of the grafted CA-RO membranes

**Figure 8:**
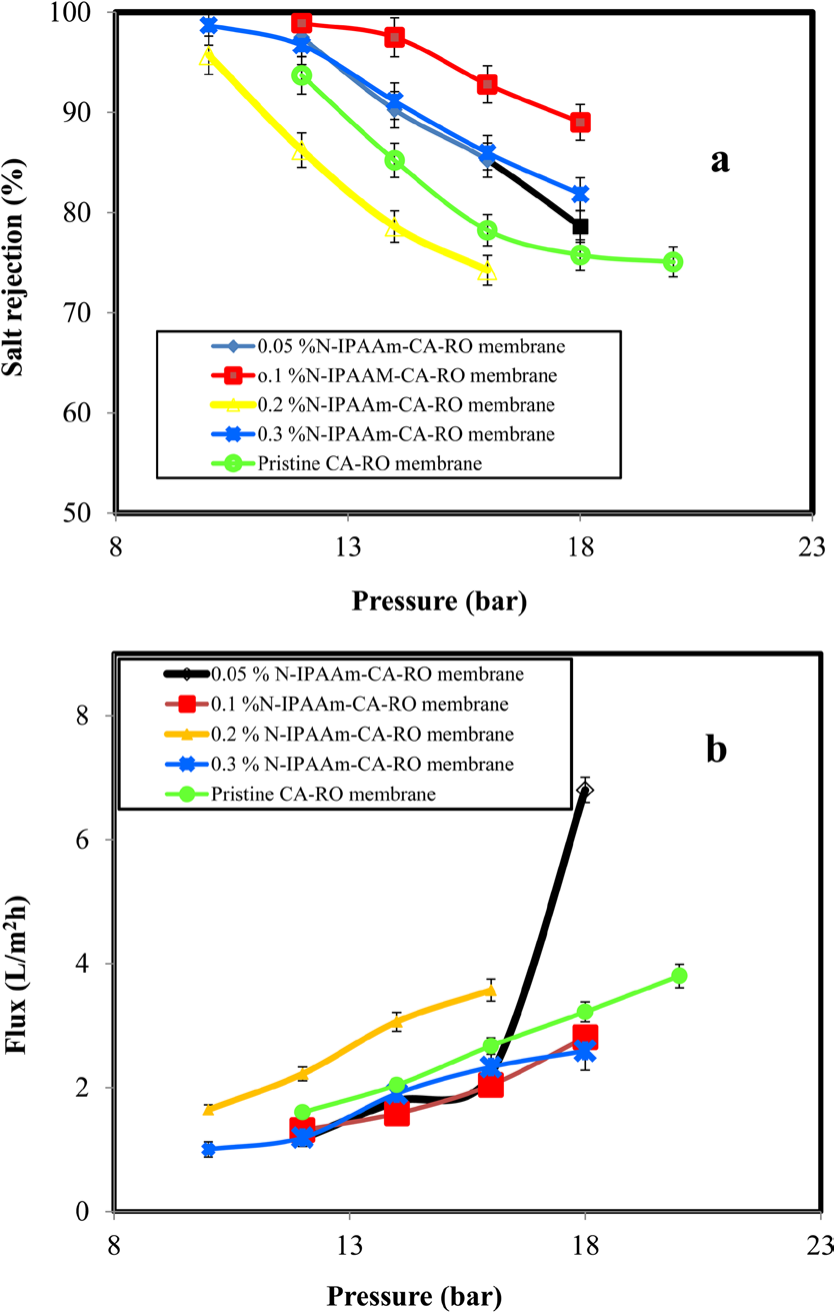
(**a**) Salt rejection and (**b**) Water flux of different grafting membrane concentrations.

The salt rejection and permeate water flux of the treated CA-RO membranes with different grafting concentrations are evaluated and the results are shown in Fig. 8(a,b). The 0.05% grafted, 0.1% grafted, 0.2% grafted, and 0.3% grafted membranes have salt rejection values of 97.5%, 98.9%, 97.9% and 96.7%, respectively at the 12 bar (Fig. 8a). Interestingly, conducting the measurements at 18 bar, the salt rejection values are 78.6%, 89%, 88%, 81.84%, respectively. The salt rejection results inversely proportional to increasing pressure. The small pores in the grafted membrane exhibit better salt rejection than the dense skin untreated analogue^39^. On the other hand, the permeate water flux of the above-mentioned grafted samples conducted at 12 bar are, respectively, 1.18 L/m^2^h, 1.31 L/m^2^h, 1.03 L/m^2^h and 1.19 L/m^2^h as presented in Fig. 8(b). However, conducting the tests of the previously mentioned grafted membranes at 18 bar, the water fluxes are improved to 6.8 L/m^2^h, 2.81% L/m^2^h, 2.9 L/m^2^h and 2.59 L/m^2^h, respectively. This result could be explained in terms of either the formation of compact layer or the chain pores blocking by the grafting monomer molecules^28,40^.

### Salt rejection and water flux performance of the grafted/crosslinked CA-RO membranes

**Figure 9:**
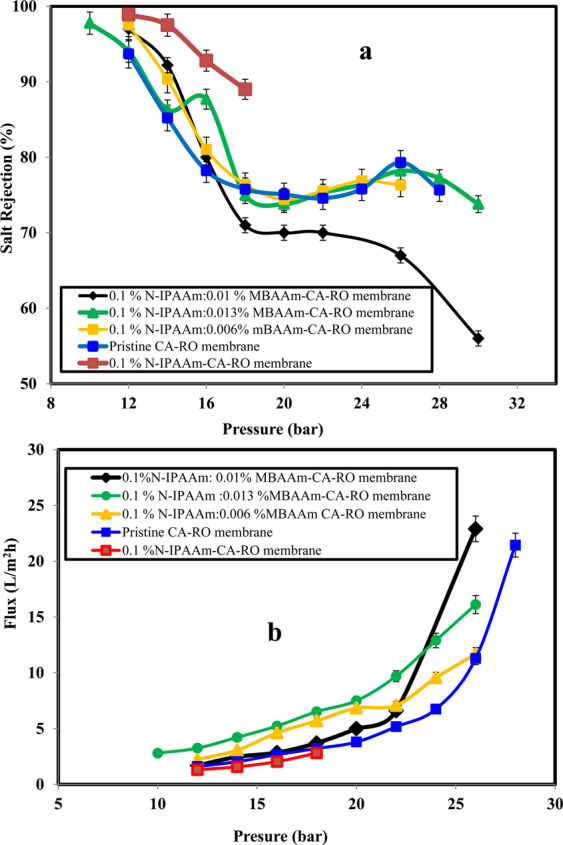
(**a**) Salt rejection and (**b**) water flux of different grafting/crosslinking membrane concentrations.

Based on the above results of different grafting concentrations, the relatively efficient 0.1 wt % N-IPAAm-grafted membrane is selected for further MBAAm crosslinking-performance studies as displayed in Fig. 9. Salt rejection results conducted at 12 bar of (0.1% grafted-/0.006 wt % crosslinked-), (0.1 wt % grafted-/0.01 wt % crosslinked-) and (0.1 wt % grafted-/0.013 wt % crosslinked) CA-RO membranes are 97.53, 97.0, and 94%, respectively as depicted in Fig. 9(a). Noticeably, the experiments at 26 bar for the same mentioned grafted/crosslinked CA-RO membranes, the salt rejection values are found to be 76.3, 67 and 78.2%, respectively. Likewise, the salt rejection of 0.1 wt % grafted/0.013% wt crosslinked-CA-RO membranes at 10 bar is 97.77% as shown in Fig. 9(a). It is noted that grafting/crosslinking process improved the salt rejection and this result could be explained in terms of increasing the crosslinker-CA matrix interaction, which subsequently increase the free volume throughout the surface, and improve water transport^40^. In addition, crosslinker may reduce the degree of crystallinity of CA and thus, facilitate water diffusion^41,42^.

Otherwise, conducting the salt rejection for 0.1 wt % grafted/0.02 wt % crosslinked CA-RO membrane leads to surface blocking and no water passage is noticed at 12 bar. However, water transport is only observed starting at 24 bar. For this specific membrane, salt rejection value is 99% and decreases to 77% on further increase of the operating pressure up to 34 bar. On the other hands, the above grafted/crosslinked membranes appear water flux at 12 bars of 2.23 L/m^2^h, 1.7 L/m^2^h and 3.25 L/m^2^h, respectively as illustrated in Fig. 9(b) and the water flux results at 26 bar are 11.67 L/m^2^h, 22.9 L/m^2^h and 16.12 L/m^2^h, respectively.

**Figure 10:**
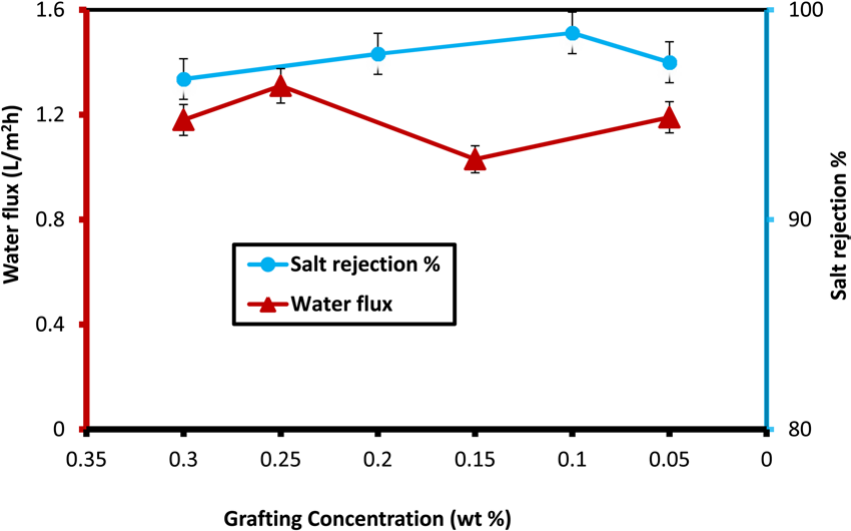
Salt rejection and water flux of different grafting concentrations at 12 bar.

Figure 10 summarizes the results of the water flux and salt rejection versus different concentrations of grafting N-IPAAm onto the CA-RO membrane surface at 12 bar. It can be concluded that the 0.1 wt % N-IPAAm/CA-RO membrane produced the highest salt rejection of 98.9% with water flux 1.3 L/m^2^h. On the other hand, the salt rejection and water flux at 26 bar of 0.1 wt % grafted/0.013 wt % crosslinked CA-RO membrane are 78.2% and 16.12 L/m^2^h, respectively.

## Conclusions

CA-RO membrane was successfully prepared by the phase inversion process and the modification of its surface was conducted by grafting process using different concentrations of N-IPAAm. AFM images of the pure CA-RO and the 0.1 wt% N-IPAAm-grafted CA-RO showed that the surface roughness of the former was 42.99 nm and decreased to 11.6 nm in the latter sample. The AFM images showed that the surface roughness of the grafted/crosslinked CA-RO sample was 27.1 nm. The contact angle of pristine CA-RO membrane was 66.28° and declined to 49.7° by the treatment with 0.1% grafting agent. The optimum grafted/crosslinked composition of N-IPAAm /MBAAm was 0.1 wt % / 0.013 wt % at operating pressure up to 30 bar and produced a salt rejection of 94% and water flux of 3.2 L/m^2^h. It was obtained that 0.1 wt % grafted/0.013 wt % crosslinked CA-RO membrane at 26 bar produced salt rejection and water flux of 78.2% and 16.12 L/m^2^h, respectively.
